# The Need for Formally Defining “Modern Medicine” by Means of Experimental Design

**DOI:** 10.3389/fgene.2016.00060

**Published:** 2016-04-20

**Authors:** Frank Emmert-Streib, Lauri Tuomisto, Olli Yli-Harja

**Affiliations:** ^1^Predictive Medicine and Analytics Lab, Department of Signal Processing, Tampere University of TechnologyTampere, Finland; ^2^Computational Systems Biology, Department of Signal Processing, Tampere University of TechnologyTampere, Finland

**Keywords:** personalized medicine, precision medicine, genomics, computational biology, biostatistics, biomedical data science

There is general agreement that the manner medicine is currently practiced is not adequate for addressing all health relevant issues in a way that would be most beneficial for all patients and that we need a new *modern medicine*. Even candidates for such a modern medicine have been recently nominated in the form of (I) personalized medicine, (II) precision medicine, (III) P4 medicine, (IV) P5 medicine, and (V) stratified medicine, which we will review briefly. The purpose of our opinion paper is to emphasize the urgent need for a formal definition in terms of the experimental design of modern medicine in order to deal efficiently with the opportunities provided by high-throughput genomic data and that all the current candidate programs mentioned above are lacking such a formalization.

In order to reveal these shortcomings and to see the need for revised formulations, we provide in the following a brief account on the proposed approaches (I–V). According to the Committee on a Framework for Development a New Taxonomy of Disease, National Research Council (2011):

‘Precision medicine’ refers to the tailoring of medical treatment to the individual characteristics of each patient. It does not literally mean the creation of drugs or medical devices that are unique to a patient, but rather the ability to classify individuals into subpopulations that differ in their susceptibility to a particular disease, *in the biology and/or prognosis of those diseases they may develop*, or in their response to a specific treatment. Preventive or therapeutic interventions can then be concentrated on those who will benefit, sparing expense and side effects for those who will not.

For personalized medicine the committee gives the exact same definition, with the difference of omitting the italic parts in the above statement. Furthermore, it mentions that:

Although the term ‘Personalized Medicine’ is also used to convey this meaning, that term is sometimes misinterpreted as implying that unique treatments can be designed for each individual.

P4 medicine (Personalized, Predictive, Preventive, Participatory) (Weston and Hood, 2004) has been proposed by Leroy Hood. Its basic idea is founded on systems biology and the fact that the function of genes and gene products unfolds via different types of interaction networks, e.g., the protein interaction network or the transcriptional regulatory network of cells and the intra network interactions among these networks (Kauffman, 1969). Hood emphasizes the need for a cross-disciplinary environment because only this enables an effective integration of biology, technology and computation/mathematics in order to establish P4 medicine. Unfortunately, no quantitative approach is defined. Interestingly, further qualitative extensions to P4 medicine have been suggested by Gorini and Pravettoni (2011) named P5 medicine, where the additional “P” represents *psycho-cognitive aspects* and Khoury et al. (2012) arguing that the population perspective to the system needs to receive more emphasize. Finally, stratified medicine aims, compared to the above programs, to more modest goals, trying to connect patient's subpopulations via clinical biomarkers to therapeutic responses (Trusheim et al., 2007). Although, also this program describes only a qualitative approach, it is clear that the practical carrier of this approach are *clinical biomarkers* opening the door for all types of high-throughput Omics and imaging technologies. However, this assumes biomarkers are well defined. Here it is important to emphasize that the National Biomarker Development Alliance reveiced recently the definition of “a biomarker” because it has been realized that previous definitions contain systematic shortcomings.

From the brief outline of the five modern medicine programs above, we think it is fair to assert that each of them is *qualitative* in nature, in the sense that it would not entail a *quantitative* definition in statistical terms of the required experimental design (Hinkelmann and Kempthorne, 2008). However, this is a tremendous shortcoming because each of these modern medicine programs will be based on genomics data, which can only be utilized beneficially if one specifies the way they should be *used* with sufficient detail. Particularly, this requires the full specification of every step from the generation of the data down to their analysis, in order to ensure the optimal value for the patients and all statistical necessities, including the robustness and the reproducibility of the obtained results (Peng, 2011).

For instance, although the above five novel approaches to medicine are based on Omics data, none is explicit enough to identify the population under investigation for a particular problem category. As an example, randomized clinical trials, the gold standard of the “old medicine” use statistical hypothesis testing to decide about the effect of a new treatment or drug w.r.t. clinical outcome endpoints, whereas patients correspond to samples in a trial. Will the new approaches to medicine involve statistical hypothesis testing too, we think so, but what is then a sample? If it is a patient then what is the difference to our current approach? If one uses clinical biomarkers to stratify patients into smaller subpopulations (subgroups) of patients, will there be enough patients in these strata to allow for enough power of a statistical test? For orphan diseases this is certainly questionable because the number of cases is limited. As an extreme example consider Ribose-5-phosphate isomerase deficiency with a single diagnosed patient. Would any of the five new medicine programs provide a solution for this, or are such disorders excluded? If they are excluded, were to draw the line for exclusion? Would this imply that for the left out disorders we need yet another new medicine 3.0? These are just a few arguments that come to mind for which we cannot find satisfactory answers in the proposed agendas. Overall, we think that further qualitative refinements of such programs are not fruitful because they would fuel further confusion rather than providing clarity.

We think there are two major reasons for these problems. First, each of the five new medicine paradigms has been proposed *before* big genomic, clinical and electronic health data have become widely available in an affordable manner. For this reason it was not feasible to base a discussion on a long lasting and practical *hands-on* experience. Even now we haven't fully reached this stage yet, but at least to some extend, several such data sets of such sort are available, most notably publicly provided by The Cancer Genome Atlas (TCGA) (The Cancer Genome Atlas Research Network, 2008). This deficiency of data, and the accompanying analysis expertise, introduced inevitably some uncertainty in the formulation of the proposed modern medicine programs and these gaps are becoming presently more and more obvious and severe the more data are actually becoming tangible. One expression of this confusion could be observed during the 11th Annual Personalized Medicine Conference 2015 held at the Harvard Medical School in Boston, a prime conference in personalized medicine, where the speakers could not present a coherent view, e.g., in comparison with non-personalized medicine approaches. Second, classically, medicine is the domain of practicing medical doctors and clinicians, but there are well-known documented problems in the understanding of statistical methodology by these professions, even for comparably simple statistical analysis tasks (Casscells et al., 1978; Manrai et al., 2014). Relating back to large-scale Omics data, the required proficiency level for such data is certainly manyfold higher and for this reason we think it is fair to assume that an assigned committee should involve experts from all relevant fields, including computational biology and biostatistics, for the formulation of a modern medicine program. In this context we would like to note that this was, e.g., not the case for the Committee on a Framework for Development a New Taxonomy of Disease, National Research Council (2011).

We believe that modern medicine, in contrast to the classical fields of medicine, should be a truely interdisciplinary field and for this reason requires expertise from areas other than medicine in a significant and profound manner. The expertise that comes from the outside of medicine, needs to be broadly centered around skill sets of the experimental design of all types of relevant big data, including genomic, clinical and health record data, as covered for instance by computational biology, biostatistics and medical informatics. This implies that every attempt for proposing a new paradigm should be best based on a consortium of experts covering all relevant fields, including the above mentioned quantitative subjects. Furthermore, it seems impossible at the current time to be able to achieve a final formulation in a one-step process. For this reason a stepwise procedural approach should be taken allowing the adjustment of such a formulation over time by taking new developments into account. In this way, the formulation of modern medicine could evolve naturally.

The current state of this problem brings back when biology transformed into a data-driven science at the beginning of the millennium. Since then, computational biology and related fields are providing an integral part by forming an inseparable unit with biology, not only for providing services, but for leading the field. We think that a similar transformation is needed for medicine establishing computational medicine as an integral part of it (Shah and Tenenbaum, 2012). Only in this way the experimental design needs of modern medicine are given full quantitative considerations to make an efficient and integrative use of all newly available biotechnologies producing data for providing the best services for the patients.

## Author contributions

FES conceived the study. All authors wrote the article and approved the final version.

## Funding

FES would like to thank TUT for financial support.

## Conflict of interest statement

The authors declare that the research was conducted in the absence of any commercial or financial relationships that could be construed as a potential conflict of interest. The reviewer (AA) and Handling Editor declared their shared affiliation, and the Handling Editor states that the process nevertheless met the standards of a fair and objective review.
